# Fungal artificial chromosomes for mining of the fungal secondary metabolome

**DOI:** 10.1186/s12864-015-1561-x

**Published:** 2015-04-29

**Authors:** Jin Woo Bok, Rosa Ye, Kenneth D Clevenger, David Mead, Megan Wagner, Amanda Krerowicz, Jessica C Albright, Anthony W Goering, Paul M Thomas, Neil L Kelleher, Nancy P Keller, Chengcang C Wu

**Affiliations:** Department of Medical Microbiology and Immunology and Bacteriology, University of Wisconsin at Madison, Madison, WI USA; Intact Genomics, Inc., St Louis, MO USA; Proteomics Center of Excellence, Northwestern University, Evanston, IL USA; Lucigen Corporation, Middleton, WI USA; Department of Chemistry, Northwestern University, Evanston, IL USA; Department of Molecular Biosciences, Northwestern University, Evanston, IL USA

**Keywords:** Fungal artificial chromosome (FAC), Functional genomics, Secondary metabolite (SM) gene clusters, Natural product discovery

## Abstract

**Background:**

With thousands of fungal genomes being sequenced, each genome containing up to 70 secondary metabolite (SM) clusters 30–80 kb in size, breakthrough techniques are needed to characterize this SM wealth.

**Results:**

Here we describe a novel system-level methodology for unbiased cloning of intact large SM clusters from a single fungal genome for one-step transformation and expression in a model host. All 56 intact SM clusters from *Aspergillus terreus* were individually captured in self-replicating fungal artificial chromosomes (FACs) containing both the *E. coli* F replicon and an *Aspergillus* autonomously replicating sequence (AMA1). Candidate FACs were successfully shuttled between *E. coli* and the heterologous expression host *A. nidulans*. As proof-of-concept, an *A. nidulans* FAC strain was characterized in a novel liquid chromatography-high resolution mass spectrometry (LC-HRMS) and data analysis pipeline, leading to the discovery of the *A. terreus* astechrome biosynthetic machinery.

**Conclusion:**

The method we present can be used to capture the entire set of intact SM gene clusters and/or pathways from fungal species for heterologous expression in *A. nidulans* and natural product discovery.

**Electronic supplementary material:**

The online version of this article (doi:10.1186/s12864-015-1561-x) contains supplementary material, which is available to authorized users.

## Background

Secondary metabolites (SMs), also known as natural products, are a structurally diverse group of compounds with varied and important biological activities. Fungi are prolific producers of these compounds, which can be classified as polyketide, non-ribosomal peptide, terpene or molecules of mixed heritage (e.g. polyketide-non-ribosomal peptide hybrids). Well-known fungal secondary metabolites include the antibiotic penicillin from *Penicillium chrysogenum*, the immunosuppressant cyclosporine from *Tolypocladium inflatum*, and the cholesterol-lowering agent mevinolin (a.k.a. lovastatin) from *A. terreus*. With over 5 million predicted fungal species [1] and dozens of secondary metabolite (SM) clusters per species [2], the number of yet undiscovered SMs is quite large. Indeed, recognition of SM clusters and other valuable attributes within the fungal genome led to the DOE Joint Genome Institute’s 1000 Fungal Genomes large-scale sequencing project [3].

Despite the abundance of available fungal genetic sequence data highlighting an enormous abundance of SM clusters, many remain ‘silent’ in laboratory growth conditions and require genetic manipulation to be expressed. Several approaches have been taken to ‘turn-on’ SM clusters with some success. These include over-expressing cluster specific transcription factors or enzymatic genes, deleting or over-expressing chromatin modifying genes, over-expressing trans-acting activators or deleting trans-acting inhibitors [4-7]. These molecular machinations, however, only work for genetically amenable fungi of which there are remarkably few. This latter point has led to the use of SM workhorses, most commonly members of the genus *Aspergillus* or yeast, for expression of heterologous SM genes [8-12].

Expression of a heterologous cluster in fungi is one approach to identify the encoded SM and the biosynthetic genes responsible for its biosynthesis, but this is not a trivial undertaking. Recently, this approach has been reported for synthesis of the *A. terreus*-encoded compounds geodin and asperfuranone using *A. nidulans* as the heterologous host [9,10]. *A. nidulans* was also used to heterologously express a dermatophyte-derived gene cluster responsible for the synthesis of neosartoricin B [12]. These studies utilized yeast recombinatory plasmids, multiple fusion PCRs and numerous transformation events to stitch together and insert individual genes to create a single full length cluster in *A. nidulans*. These technologies require considerable effort and time to express just one heterologous cluster and have been limited in the size of the inserted cluster.

Bacterial artificial chromosomes (BACs) have been widely used for genomic DNA sequencing, positional cloning, and mapping in prokaryotes and eukaryotes including filamentous fungi [13-17]. Although large-insert DNA systems have also been applied for heterologous expression of microbial natural product biosynthetic pathways and metagenomic studies, there has been limited success reported due to technological challenges [18-20]. The challenges include but are not limited to: 1) DNA cloning bias; 2) small DNA insert size; 3) lack of advanced heterologous expression hosts and 4) insufficient high-resolution chemical and data analysis pipelines. Here we address all of these limitations through creation of a novel *Aspergillus/E. coli* shuttle fungal artificial chromosome (FAC) expression vector by utilizing unbiased Random Shear BAC technology [21] coupled with an autonomous fungal replicating element AMA1 [22], to expression of FACs in *A. nidulans,* to characterization of FAC SMs using state-of-art liquid chromatography-high resolution mass spectrometry (LC-HRMS) and a chemoinformatic analysis pipeline. We present the heretofore undiscovered *A. terreus* astechrome biosynthetic machineries as proof-of-concept of our FAC SM methodology.

## Results

### Construction of unbiased shuttle BAC library of *A. terreus* DNA and heterologous expression of SM clusters as FACs in *A. nidulans*

To develop a fungal artificial chromosome (FAC) system, we used unbiased Random Shear BACs as the basis for our technology as BAC inserts can reach up to 300 kb, and Random Shear BAC cloning generates even coverage of a fungal genome for the selection of all SM clusters within the genome [21,23]. The Lucigen pSMART BAC vector was modified to operate as a shuttle vector between *E. coli* and *A. nidulans*. The AMA1 DNA fragment, previously identified as an autonomously replicating DNA fragment from *A. nidulans* [22], was incorporated into pSMART BAC to create pSMART-BAC pyrGAMA1-4, or shuttle BAC vectors, and tested for autonomous replication in *A. nidulans* as FACs (Additional file 1: Figure S1 a, b).

**Figure 1:**
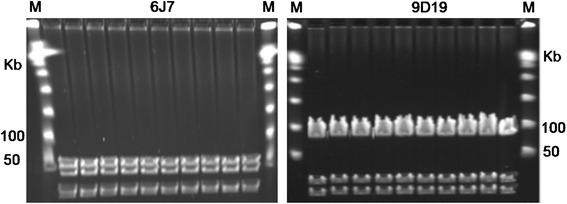
The CHEF gels of *E. coli-Aspergillus* shuttle FACs: 6J7 (cluster 44, ~112 kb) and 9D19 (cluster 23, ~100 kb) that were successfully transferred from transformed strains of *A. nidulans* back into *E. coli.* The first and last lanes (M) are DNA Lambda ladder Markers, the 2^nd^ lane on the left hand side of the gels is the control FAC used to transform *A. nidulans*, and all of other lanes are randomly selected FAC clones recovered. All control and recovered FACs were digested with *NotI*.

*A. terreus* was selected for shuttle BAC DNA library construction because it has a fully sequenced genome containing 56 annotated SM gene clusters [24]. High molecular weight genomic DNA was prepared from *A. terreus* and construction of the unbiased BAC library resulted in ~20x genome coverage of the *A. terreus* genome, or a total of 7,680 BAC clones with an average insert size of 100 kb (Additional file 1: Figure S2a, b). The BAC library was arrayed into 384-well plates and both ends of 3,840 BAC clones were sequenced. Sequence alignment of these end sequences with the *A. terreus* reference genome was used to identify SM-BAC clones or candidate FACs containing all 56 SM gene clusters (Additional file 2: Table S1). Fifteen FACs (ranging from 70 to 150 kb in size) were selected for heterologous expression and analysis through transformation into *A. nidulans* (Table 1). All were successfully transformed into *A. nidulans.* To validate the shuttle function of FACs, we also extracted five of the 15 FAC DNAs from transformed *A. nidulans* strains and successfully transformed FAC DNA back into *E. coli* (Figure 1, Additional file 1: Figure S3). This was the first demonstration of the capability of AMA1 in supporting autonomous replication (FAC) of large DNA constructs greater than 100 kb in *A. nidulans*.

**Table 1:** **Fifteen FACs chosen to transform into** ***A. nidulans***

**FAC name**	AT cluster(s)*	**PCR**	TX/μg FAC**	**Fungal strain**
4G11	21	All	123	TJW152
6J7	44	All	94	TJW153
8J11	52	One end only	36	TJW154
9O3	30	All	43	TJW155
4O12	11	All	58	TJW156
6H10	6	All	60	TJW157
8K17	19	All	139	TJW158
5N15	39	All	24	TJW159
7O19	38	All	18	TJW160
9F18	55	All	57	TJW161
6C13	41	All	94	TJW162
7P13	50	All	29	TJW163
9D19	23	All	80	TJW164
9A23	25, partial 26	All	26	TJW165
7A10	56	All	45	TJW166
Empty	No		432	TJW167

The *A. terreus* clusters they contain are indicated as well as PCR results denoting if all or part of the cluster was confirmed in the FAC clone.

*AT = A. terreus, **TX = transformants.

### LC-HRMS linked SM library screening

**Figure 2:**
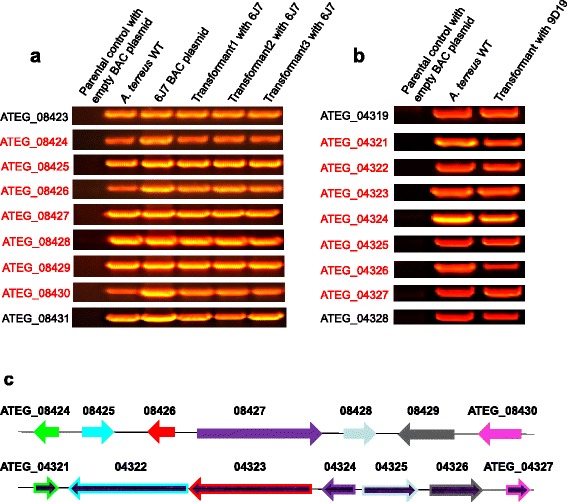
FAC transformants contain intact gene clusters confirmed by PCR. Panel **a**: Astechrome gene cluster (6J7) is present in *A. nidulans* transformed with FAC as examined by PCR. The seven astechrome genes (red) are not present in wild type *A. nidulans* but are in *A. terreus* and all *A. nidulans* 6J7 transformants. Panel **b**: Putative gene cluster 23 (9D19) is present in A. nidulans transformed with FAC 9D19 as examined by PCR. The seven cluster genes (red) are not present in wild type *A. nidulans* but are in *A. terreus* and *A. nidulans* 9D19 transformant. All of primers are listed in Additional file 2: Table S1. Panel **c** shows both Astechrome gene cluster (6J7) on the top and the cluster 23 (9D19) on the bottom.

**Figure 3:**
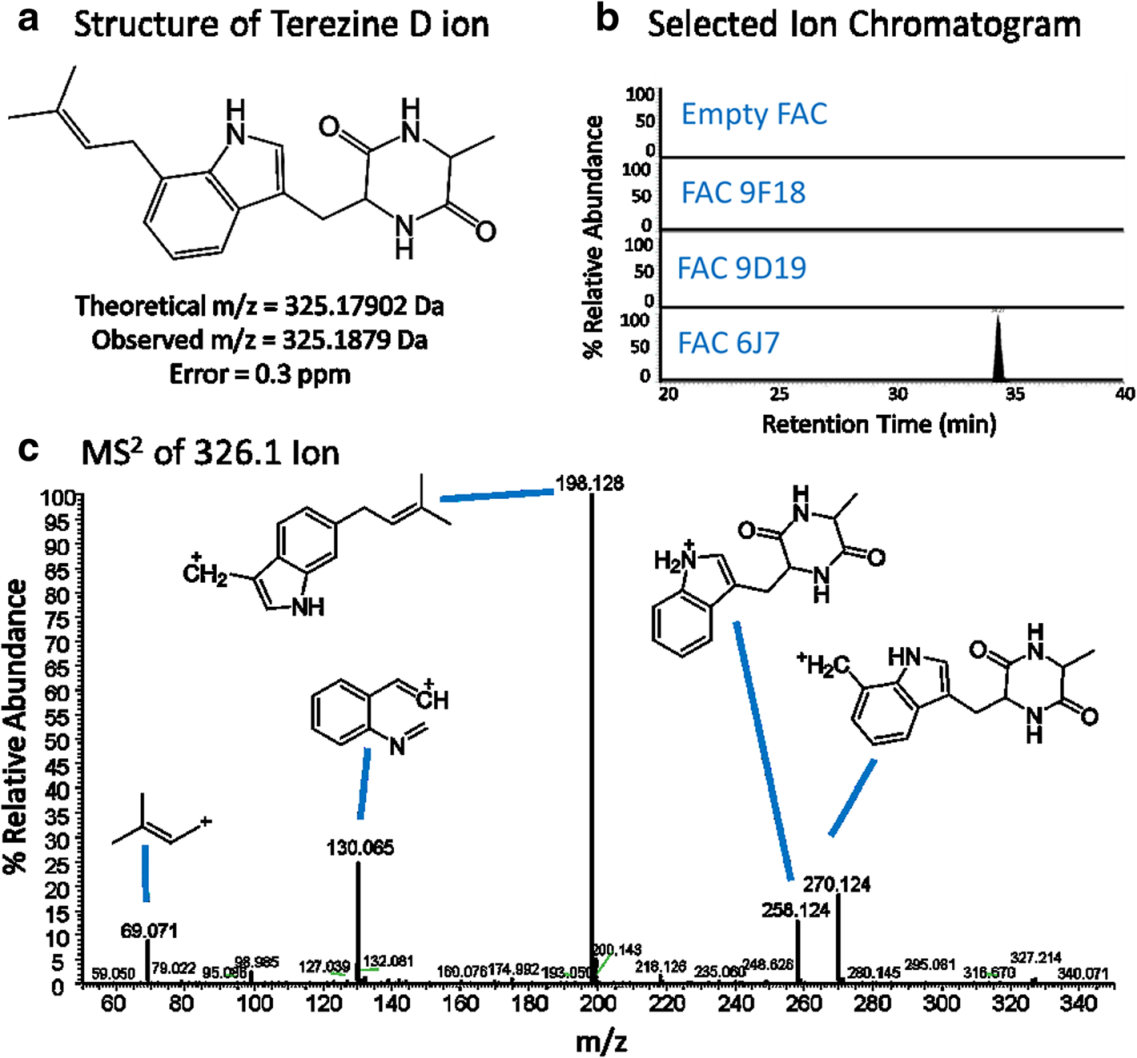
**Identification of astechrome biosynthetic precursor, terezine D.** **(a)** A compound was identified in FAC 6J7 cell extracts with exact mass matching the theoretical exact mass of terezine D, the predicted product of FAC 6J7. **(b)** Selected ion chromatograms of lysates from cells containing FAC 6J7 and all other analyzed FACs show that the compound of interest is unique to the FAC 6J7 expressing strain. **(c)** Analysis of MS^2^ fragmentation data for the identified compound lead to identification of predicted fragments of terezine D, confirming the compound’s identity.

Upon confirmation that *A. nidulans* faithfully replicated FAC DNA, *A. nidulans* FAC 6J7 strain was selected for initial proof-of-concept experiments, as it contained a cluster highly homologous to the recently characterized hexadehydroastechrome cluster in *A. fumigatus* [25]. FAC 6J7 contains seven out of the eight genes found in the corresponding *A. fumigatus* cluster (Figure 2a). The gene, *hasG*, not present in the *A. terreus* cluster, encodes for an FAD binding protein responsible for converting a prenyl to a methylbutadienyl side chain to produce hexadehydroastechrome from astechrome. FAC 6J7 metabolites were identified by analyzing organic extracts of the *A. nidulans* FAC 6J7 transformant and control *A. nidulans* using LC-HRMS. Following data acquisition, Sieve software was used for component detection and relative quantitation. When comparing FAC 6J7 extracts to control sample extracts (wild type and other FAC strains), a compound that was present only in the FAC 6J7 extract (Figure 3b) was identified as terezine D by both accurate mass (0.3 part-per-million error) and tandem mass spectrometry (MS/MS or MS^2^) (Figure 3a,c). Terezine D is a stable intermediate of astechrome biosynthesis [26].

## Discussion

**Figure 4:**
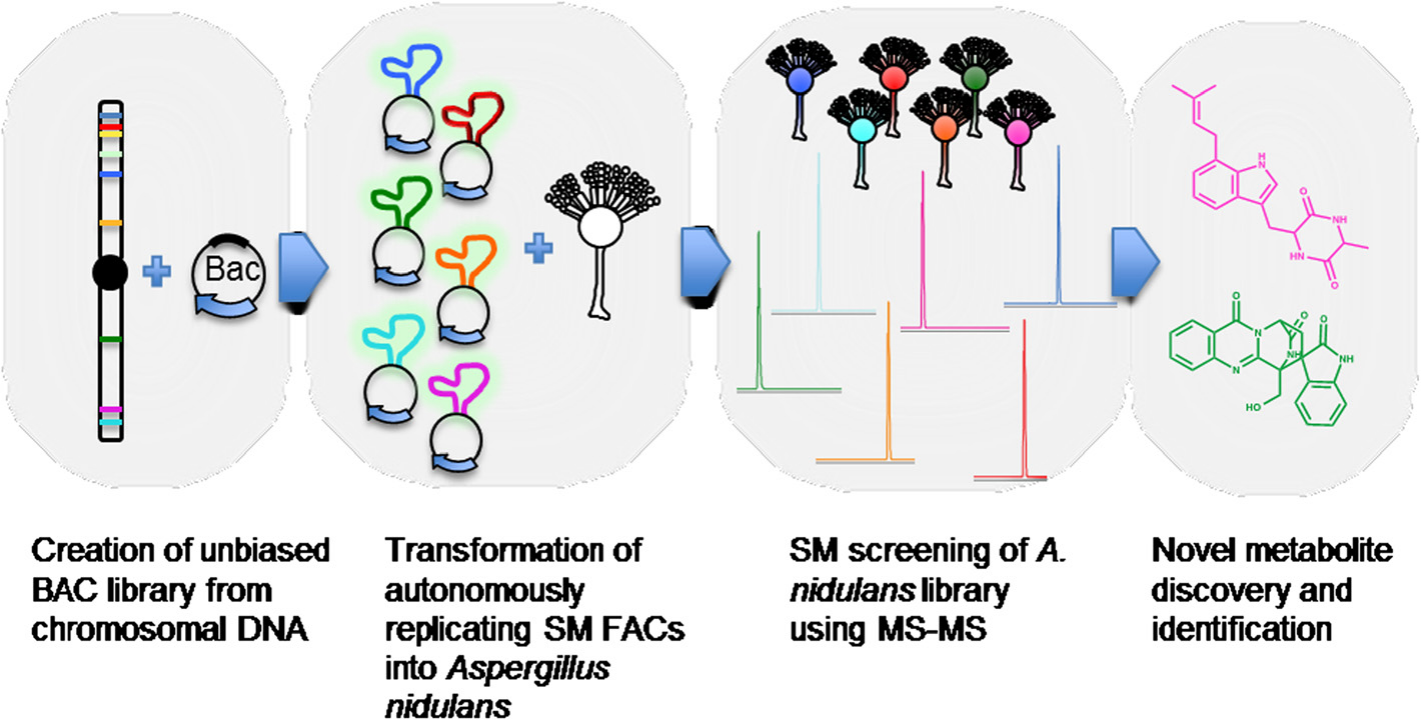
A diagram shows the workflow of cryptic SM production. High molecular weight genomic DNA from *A. terreus* was mechanically sheared and cloned into shuttle AMAI-BAC (or FAC) clones, SM FACs were selected and transformed into *A. nidulans*, transformants screened for metabolites followed by compound identification and structure elucidation.

There is an urgent need for new therapeutic agents to combat rapidly-emerging multiple drug resistant (MDR) and pan-resistant pathogens such as methicillin resistant *Staphylococcus aureus* (MRSA) and *Acinetobacter baumanii*. Filamentous fungi are prolific producers of SMs and have historically been a rich source of lead compounds for the pharmaceutical industry. Genomic sequencing data confirms that fungi contain a far greater biosynthetic capacity than has been realized to date, and thus fungi should continue to be viewed as important reservoirs for novel bioactive compounds [27-33]. In fact the number of SM cluster sequences available for characterization far outstrips our current ability to characterize each cluster. To address this post-genomic SM characterization gridlock, in this report we have demonstrated a new technology that generates a whole genome SM FAC library for expression in suitable host systems and characterization in high-throughput chemical analysis pipelines. An overview of this technology is presented in Figure 4.

To date, there does not exist an efficient heterologous expression system to cover entire SM clusters (30 – 80 kb) in a single cloning step. BAC vectors have been widely used for cloning large DNA fragments but there is no successful report of heterologous expression of fungal SM clusters. A previous attempt to introduce up to 75 Kb of fungal DNA into *Fusarium oxysporum* and *A. awamori* using an *Agrobacterium tumefaciens* transformation system yielded few transformants with large DNA inserts and furthermore, no attempts to examine stability of heterologous DNA, let alone expression, were made [34]. In order to build high quality shuttle BAC libraries for entire SM gene clusters, the cloning methods and vectors used are of extreme importance. The bias introduced by partial restriction digestion of genomic DNA results in various regions being highly under-represented, over-represented, or missing for all eukaryotic multi-cellular genomes studied, including *Arabidopsis*, *Drosophila*, rice, mouse, fungi and humans. The bias is most evident in certain regions of genomic DNA that contain highly repetitive sequences, such as centromeres and telomeres [21]. As a result, numerous clone gaps can be impossible to close, even with multiple biased partial digestion libraries and up to 50 x coverage, thus dramatically increasing finishing costs. Fungi are known to contain many SM clusters in telomeric and subtelomeric regions of the genome [7,24] and from our BAC end sequencing and reference genome alignment, we found that at least 10 of 56 SM clusters of *A. terreus* are located near telomeres and some telomeric sequences are still not complete in the whole genome sequence database (data not shown). We successfully overcame this potential bias in conventional BAC library construction through the introduction of randomly sheared genomic DNA into the FAC vectors.

To improve transformation yield and subsequent expression level of the gene clusters in a heterologous system, the BAC vector was modified into a FAC vector by inserting a fungal self-replicating element. The AMA1 sequence from *A. nidulans* is known to increase transformation efficiency up to 2000-fold compared to a traditional integrating plasmid (reviewed in [22]). The AMA1 sequence was also shown to be fully or partially functional in other filamentous fungal systems including *A. fumigatus* resulting in 10–30 copy numbers per cell and increased gene expression [22,35-40]. By introducing the AMA1 element in a BAC vector, thus creating the FAC system, we were able to introduce and express at least 150 kb heterologous DNA in *A. nidulans*. Strictly the concept of BAC itself is truly an F-plasmid, not a ‘bacterial artificial chromosome’, FAC can be justified similarly as BAC with the capability of shuttling and maintaining large DNA and potential wide applications. In addition, FAC, a shuttle BAC vector, is capable of cloning >300 kb insert DNA in *E. coli* [41]; it will be interesting to see the size limitation and stability of more FACs in *A. nidulans* as compared with the yeast artificial chromosome (YAC) in *Saccharomyces cerevisiae* [42].

The entire *A. terreus* SM genome was captured in our FAC library with 56 SM clusters (Additional file 2: Table S1). The availability of such a library is powerful, allowing us to swiftly screen the host recipient for individual SM activities. The host recipient is critical for transformation and expression properties and we found the genetic model *A. nidulans* to be an efficient host (see Methods for FAC transformation optimization). As a first assessment of FAC transformation, 15 FACs were successfully expressed in *A. nidulans* (Table 1) and, equally important, successfully transferred back to *E. coli* (Figure 1).

A FAC based on an *A. terreus* SM cluster similar to a known *A. fumigatus* hexadehydroastechrome cluster [25], was chosen for HRMS screening and characterization. *A. nidulans* FAC 6J7 yielded a unique compound, the stable astechrome intermediate terezine D (Figure 3). This was consistent with the predicted metabolite production for this gene cluster (Figure 2a). Currently our focus is on elucidating novel chemistries of two other FACs which produce metabolites with masses that are not consistent with any known fungal metabolites (data not shown), as well as measuring mRNA levels in strains expressing FACs of interest (including Fac 9D19) to confirm that all encoded genes are being correctly expressed.

We anticipate that many alternative and expanded studies will follow on from this work. For instance, in addition to the FAC end sequencing and reference genome alignment presented in this work, there are optional and potentially more economic genomic tools to identify SM containing FACs, such as pooling FACs for either PCR or Southern hybridization-based library screening of SM backbone genes. Alternatively, extracts of FAC transformants could be first screened for desired activity and then only those showing activity be subjected to FAC sequencing. These alternative approaches could be useful for those genomes which are poorly- or not sequenced. We also envision an expansion of FAC libraries to include not only *Aspergillus* spp. but also other genera. The successful expression of *Neurospora crassa* and lichen promoters in *A. nidulans* may suggest that a fair number of Ascomycetes can be used in our system, because the lichen promoters are most likely from the fungus in the symbiotic relationship, which is typically an ascomycete [43,44].

## Conclusions

The results that we have described represent significant advancements to the field of translating whole genome sequence information into functional genomics and genome biology. We report the first FAC system, which is capable to shuttle and stably maintain large (>150 kb) DNA fragments in both *E. coli* and the filamentous fungus. Our concept of a large FAC equals one intact SM gene cluster including all genes and regulatory elements within a large gene cluster and/or pathway for heterologous expression provides a route for the discovery of natural products potentially missed by traditional methods. Further analysis of the entire set of the intact SM gene clusters of *A. terreus* will deepen our understanding of the dynamics of SM gene pathways and the fungal natural products.

In summary, we have successfully created a breakthrough FAC technology that can help address the challenge of characterizing the accruing fungal SM genome data. This technology allows for the creation of a SM cluster library of a single fungal species that can be shuttled and expressed in *A. nidulans* and likely other appropriate fungal hosts in one transformation step. This will allow the detailed genetic analysis and manipulation of fungal gene clusters from a wide range of species in the future. Additionally, we have validated an analytical and statistical pipeline to confidently identify the compound(s) encoded by the SM cluster-containing FACs, resulting in the confident discovery and identification of the astechrome precursor terezine D and discovery of the astechrome biosynthetic machinery in *A. terreus*. This methodology enables the unbiased library construction of entire genomes of not only sequenced fungi but also potentially for unsequenced, and even unculturable fungi when sufficient material can be collected for DNA preparation. When combined with the high sensitivity of HRMS-based metabolomics, this technology has the potential to identify intact gene clusters and their associated SMs in fungi and other complex microbial metagenomes on a scale not previously considered feasible.

## Methods

### Construction of shuttle BAC vectors

To construct the *E. coli* and *Aspergillus* shuttle BAC vectors, the *A. nidulans* AMA1 gene fragment (5.250 kb) was blunt-ended with the DNA terminator kit (Lucigen) and cloned into the blunted-B*amHI* site immediately next to the *Aspergillus parasiticus pyrG* gene in pJW24 to form pJW24-AMA1. The 8.385 kb DNA fragment containing AMA1-*pyrG* was released from pJW24-AMA1 with N*otI* and B*ssHII* double digestion, blunt-ended and cloned into the blunted-A*paI* site of pSMART-BAC vector (www.lucigen.com). All reactions were performed with 100 ~ 200 ng of each DNA with a total 30 μL of reaction volume and 1 μL of each enzyme; DNA was purified with the QIAGEN mini kit between each step. Due to both orientation combinations of two-step blunt-end ligations, there were four versions of the autonomous replication *E. coli* and *Aspergillus* shuttle BAC vectors: pSMARTBACpyrGAMA1-4 (Additional file 1: Figure S1a). The vector sequences were confirmed by sequencing. All shuttle vectors (FAC vectors) were successfully tested for *A. nidulans* transformation (Additional file 1: Figure S1b).

### Preparation of high molecular weight *A. terreus* DNA

*Aspergillus terreus* strain ATCC2054 was chosen for this work. Different fungal starting materials were compared to test for quality of high molecular weight (HMW) genomic DNA: spores, germinated spores, protoplasts, or nuclei obtained from protoplasts. The protoplast preparation method has been described before [45]. To isolate nuclei, protoplasts were lysed with 0.5% Triton X-100 in HMW DNA preparation buffer (0.5 M Sucrose, 80 mM KCl, 10 mM Tris, 10 mM EDTA, 1 mM spermidine, 1 mM spermine, pH 9.4). The protoplasts in buffer were gently mixed, incubated on ice for 30 minutes, and the resulting nuclei pelleted at 1,800 g for 20 minutes. To prepare low melting agarose plugs of HMW DNA, the pellet (~5×10^8^) – be it of nuclei, protoplasts, germinated spores, or spores – was resuspended with the HMW DNA preparation buffer to a total volume of 0.6 mL, and an equal volume of 1% low melting agarose was then added to the buffer to a total volume of ca 1.2 mL at 45°C. This was sufficient to make 10 plugs (about 100 μL per plug) which solidified at 4°C. The plugs were then incubated at 50°C for 48 hours in 1 mL lysis buffer/plug: 0.5 M EDTA, pH 9.0, 1% lauryl sarcosine, 1 mg/mL proteinase K. Finally, the plugs were extensively washed in 10–20 volumes of the following buffers for one hour each wash: once with buffer 1 (0.5 M EDTA, pH 9.0-9.3 at 50°C), once with buffer 2 (0.05 M EDTA, pH 8.0 on ice), three times with buffer 3 (ice cold TE plus 0.1 mM phenylmethyl sulfonyl fluoride (PMSF) on ice), three times with buffer 4 (ice cold TE on ice) and finally all plugs were stored in TE at 4°C. In order to estimate the size and yield of extracted DNA, plugs were assessed using pulsed field gel electrophoresis (PFGE) (Bio-Rad CHEF Mapper, Hercules, CA). The final quality-check condition for the HMW genomic DNA was 6 V/cm, 10 sec to 1 min switch time for 12–16 hours at 14°C by PFGE, along with appropriate HMW size markers [46]. The highest quality and quantity of HMW genomic DNA was obtained from the protoplast preparation (Additional file 1: Figure S2a).

### Construction of unbiased shuttle BAC library of *A. terreus* DNA

The HMW genomic DNA obtained from the protoplast preparation ranged from 20 ~ 200 kb. The HMW DNA from three plugs was end-repaired with the DNA terminator kit (www.lucigen.com) in a total volume of 500 μL with 10 μL of the end repairing enzymes which were heat inactivated (70°C, 15 min). T he resulting DNA was ligated with *BstX*I adaptors (10 μL of 100 μM each) in a total volume of 700 μL consisting of a ligation reaction of 10 μL ligase (2 U/μL, Epicenter). Gel-fractionated DNA fragments ranging from 100 to 200 kb were purified by PFGE. Purified large DNA fragments (about 100 μL 1–3 ng/μL) were ligated into the cloning-ready BAC *BstX*I shuttle vector (also called pSMARTBACpyrGAMA3) at 16°C for ~18 hours. Next, the ligated DNA mixture was electroporated into competent *E. coli* cells (BAC-Optimized *E. coli* 10G Replicator Cells, Lucigen). Small-scale ligations and transformations (1 μL DNA per 20 μL cells) were used to judge the cloning efficiency. The insert sizes of about 50 BAC clones were determined and confirmed to include inserts of about 100 kb (Additional file 1: Figure S2b). Once the suitability of the ligated DNA was confirmed, large-scale ligations and transformations were conducted to achieve at least 7,680 clones for colony picking (20 X 384-well plates) for the unbiased shuttle BAC library.

### BAC end sequencing, and select SM cluster-containing candidate FAC clones

BAC-end sequences of 3,840 clones from the unbiased Random Shear BAC library of *A. terreus* were completed by the Sanger BigDye sequencing method. The software Phred was used for base calling and sequence trimming. Vector masking was achieved using the DNAStar SeqMan Pro software package. The BAC end sequences were aligned against the *A. terreus* reference genome sequence by blastn http://www.broadinstitute.org/annotation/genome/aspergillus_group/Blast.html;jsessionid=20A2ECF0FCDB84CC880624664797EEF8.route980?sp=Sblastn; All 56 SM clusters-containing candidate FAC clones were successfully identified based on the FAC end sequence flanking one end of a SM cluster and the other FAC end sequence flanking the other end of the same SM cluster (data not shown).

### Microbial strains and culture conditions

The parental strain RJW256 (*pyrG89, pyroA4, Δnku70::argB, ΔST::afpyrG, veA1*) was obtained by a sexual cross between LO4641 (*riboB2, pyroA4, ΔST::AfpyrG, ΔAN7909::afpyrG, Δnku70::argB, veA1*) and RJW113.5 *(ΔveA::argB, pyrG89*). RJW256 was transformed with FAC plasmids as shown in Table 1 to produce FAC recombinant strains. *ΔST::AfpyrG* indicates that the entire endogenous sterigmatocystin gene cluster was removed from *A. nidulans.*

For antimicrobial activity tests, we used *A. nidulans RDIT9.32, A. fumigatus 293*, *Candida albicans*, *Pseudomonas aeroginosa PAO1*, *Bacillus cereus U85*, and *Micrococcus luteus* strains. All of the fungal and bacterial strains were maintained as frozen glycerol stocks at −80°C. Fungal strains were grown at 37°C on glucose minimal medium (GMM, [45]) and bacterial strains were cultured on tryptic soy broth medium.

### *A. nidulans* transformation and the recovery of SM cluster-containing FACs

A modified PEG-calcium based transformation method was applied to improve transformation yield because our published methods [45] did not work well with the 100 kb FAC vectors. The method was modified as follows: 200 μL containing 10^7^*A. nidulans* RJW256 protoplasts mixed with 2 μg FAC DNA was gently placed over 200 μL of 30% PEG 4,000 with 50 mM CaCl_2_ in 1.5 mL centrifuge tube. The centrifuge tube with protoplasts was incubated 30 min on ice. After centrifuging the incubated mixture for 5 min at 250 × g, the solution was gently mixed using an autopipette. This mixture was then incubated for 10 min at room temperature before 1 mL of sorbitol-Tris–HCl-CaCl_2_ (STC: 1.2 M sorbitol, 10 mM Tris–HCl, 10 mM CaCl_2_ pH7.5) buffer was added and gently mixed into the solution. After transferring the mixture into a 13 mL tube, an additional 5 mL of STC was added into the tube and gently mixed. One mL of this final solution was distributed onto regeneration media to obtain transformants.

*A. nidulans* FAC transformants (Table 1) were maintained on culture plates for three generations for phenotype and chemical screening. For FAC recovery, we prepared ~0.3 mL of 10^6^/mL protoplasts from *A. nidulans* FAC strains and FAC DNA was isolated by the common alkali lysis method, and resuspended in 10 μL of TE. One microliter of recovered DNA was re-transformed back into *E. coli* cells (BAC-Optimized *E. coli* 10G Replicator Cells, Lucigen).

### Fungal genomic DNA extraction

Fungal DNA was extracted from lyophilized mycelia using previously described techniques [47] to perform PCR reaction with primers listed in Additional file 3: Table S2.

### Antimicrobial screening

A disc-diffusion method [48] was used for antibiotic activity-guided screening. One plate per each *A. nidulans* FAC strain was inoculated on solid GMM and incubated for seven days at 37°C. Subsequently, the entire contents of the plates were collected and lyophilized for 48 hours. Samples were then pulverized with mortar and pestle prior to the addition of 10 mL of methanol. Air-dried methanol extracts were dissolved in 150 μL methanol for activity testing. Media preparation for antibacterial assays were performed as previously described [45]. For antifungal assays, 10^6^ spores mentioned in the section above were embedded in 5 mL soft GMM agar (0.75% agar) and overlaid on solid GMM. Ten μL out of the 150 μL methanol extract above was loaded on a 1 cm diameter paper disc for each assay. Assay plates were incubated for 24 to 48 hour at 37°C and observed for antimicrobial activity.

### LC-HRMS analysis

Five plates for *A. nidulans* FAC strain 6J7 were inoculated on solid GMM and incubated for seven days at 37°C. Subsequently, the entire contents of the plates were collected and lyophilized for 48 hours. Samples were then pulverized with mortar and pestle prior to the addition of 10 mL of methanol. Air-dried methanol extracts were then further extracted with organic solvent (chloroform:methanol:ethylacetate = 8:1:1). Organic extracts were evaporated to dryness and stored at −20°C until analysis.

Organic extracts obtained were resuspended in methanol to a final concentration of 2 μg/μL. For each analysis, 40 μg of sample was loaded onto a Luna C18 column (150 mm × 2 mm; 3 μm particle size) (Phenomenex, Torrance, CA). Chromatography was performed using an Agilent 1150 LC system (Agilent, Santa Clara, CA) at a flow rate of 200 μL/min. The following gradient was employed (Buffer A: water with 0.1% formic acid, Buffer B: acetonitrile with 0.1% formic acid): time 0 min, 2% B; 35 min, 70% B; 54 min, 98% B. A 1:7 split was employed post-column, resulting in a flow rate of 25 μL/min being directed to the mass spectrometer. A Q-Exactive mass spectrometer (Thermo Fisher Scientific, Waltham, MA) was used for MS analysis with the following settings: capillary temperature 275°C, sheath gas 4 (arbitrary units), spray voltage 4.2 kV. Full MS spectra were acquired at 35,000 resolution for the mass range *m/z* 200 to 1500 for all samples. Following each full MS scan, the top 5 most intense ions were selected for a dependent MS^2^ scan. MS^2^ was conducted using higher-energy collisional dissociation (HCD) with a normalized collision energy of 30%. Three biological replicates of FAC 6J7 extracts were prepared and analyzed in technical duplicate, followed by the data workup described below.

### Data analysis, informatics, and software

The SIEVE software suite (Thermo Fisher Scientific, Waltham, MA) was used for component detection and relative quantification of ions produced by electrospray during small molecule LC-HRMS. Component detection was performed using a mass tolerance of 10 part-per-million (ppm) and a retention time window of 2.5 min. A minimum intensity of 5×10^6^ was selected as the threshold for defining a peak as a component. For each component, a selected ion chromatogram was created and the integrated intensity of the peak was calculated. Peak areas were normalized based on total ion current. To increase statistical power and confidence of the final analysis, the procedure adopted here involved a decoy approach to multiple hypothesis testing. Specifically, the replicate data FAC 6J7 was subjected to a uniqueness filter against processed LC-HRMS data generated from a control group of strains containing empty vectors, as well as 13 other strains containing a variety of other FACs with unique genetic content. For dereplication, all components were initially searched against a targeted accurate mass database consisting of known fungal metabolites produced by *A. nidulans* and *A. terreus* using a mass tolerance of 3 ppm. A dozen of these known compounds were present at consistent levels in nearly all samples, and were monitored to rapidly identify highly perturbed systems. All components were also searched against a comprehensive accurate mass database consisting of over 13,000 known fungal secondary metabolites. This fungal database was prepared using Antibase [49], Dictionary of Natural Products [50], as well as additional fungal natural products found in the literature [51,52].

## Additional files

Additional file 1: Figure S1. Panel a: FAC vectors: pSMARTBACpyrGAMA1 ~ 4, each has two N*ot* I sites (N) flanking the cloning site (B, B*st* XI) within the BAC end sequencing primers SP6 and T7. In addition to the chloramphenicol resistance gene (*camR*), loxP and cos sites, *pyrGA* represents the *pyr*G gene from *Aspergillus parasiticus*, AMA1 is the replication origin of fungal artificial chromosome, genes *par*A and *par*B are for active partitioning and gene *sop*C is to ensure that each daughter cell gets a copy of the shuttle BAC plasmid, gene *rep*E is for BAC plasmid replication and regulation of copy number, and oriV is the BAC replication origin. Panel b: *A. nidulans* FAC transformants using pSMARTBACpyrGAMA3 vector*.* **Figure S2.** Preparation of HMW genomic DNA from *A. terreus* and random shear FAC cloning results. Panel a: *A. terreus* HMW genomic DNA ranging from 20-200 kb. Panel b: CHEF gel electrophoresis and *Not*I digestion of random selected FAC clones, the average insert size was estimated at ~110 kb. M, Lambda ladder Marker. **Figure S3.** Three additional CHEF gels of *E. coli-Aspergillus* shuttle FACs that were successfully transferred from transformed strains of *A. nidulans* back into *E. coli.* The examples of recovered FAC clones shown here include 9O3 (cluster 30, ~100 kb), 9A23 (cluster 25, ~80 kb), and 7A10 (cluster 56, ~90 kb) from top to bottom panel. The first and last lanes are DNA Lambda ladder Markers, the 2^nd^ and 3^rd^ lane(s) on the left hand side of the gels is the control FAC used to transform *A. nidulans*, and all of other lanes are randomly selected FAC clones recovered. All control and recovered FACs were digested with *Not I*. **Figure S4.** Antibiotic activity test of 14 FAC clones. Ten μL out of 150 μL methanol extract from FAC transformants cultured on GMM plate for 7 days at 37°C was loaded on small disc (diameter: 1 cm) for antimicrobial activity test against *Aspergillus* spp., *Candida albicans, Bacillus cereus*, *Micrococcus luteus* and *Pseudomonas aeruginosa*. Antibiotic activity was observed against *Bacillus cereus* with two FAC extracts*.*
Additional file 2: Table S1. BAC clones covering 56 SM clusters identified by both BAC end sequences. Additional file 3: Table S2 PCR primers used in this study.
