# Supplementary material for: Fungal artificial chromosomes for mining of the fungal secondary metabolome
Source: BMC Genomics. 2015 Apr 29;16(1):343. doi: 10.1186/s12864-015-1561-x (PMC4413528; doi:10.1186/s12864-015-1561-x)
Supplement: Additional file 1: Figure S1. — Panel a: FAC vectors: pSMARTBACpyrGAMA1 ~ 4, each has two Not I sites (N) flanking the cloning site (B, Bst XI) within the BAC end sequencing primers SP6 and T7. In addition to the chloramphenicol resistance gene (camR), loxP and cos sites, pyrGA represents the pyrG gene from Aspergillus parasiticus, AMA1 is the replication origin of fungal artificial chromosome, genes parA and parB are for active partitioning and gene sopC is to ensure that each daughter cell gets a copy of the shuttle BAC plasmid, gene repE is for BAC plasmid replication and regulation of copy number, and oriV is the BAC replication origin. Panel b: A. nidulans FAC transformants using pSMARTBACpyrGAMA3 vector. Figure S2. Preparation of HMW genomic DNA from A. terreus and random shear FAC cloning results. Panel a: A. terreus HMW genomic DNA ranging from 20-200 kb. Panel b: CHEF gel electrophoresis and NotI digestion of random selected FAC clones, the average insert size was estimated at ~110 kb. M, Lambda ladder Marker. Figure S3. Three additional CHEF gels of E. coli-Aspergillus shuttle FACs that were successfully transferred from transformed strains of A. nidulans back into E. coli. The examples of recovered FAC clones shown here include 9O3 (cluster 30, ~100 kb), 9A23 (cluster 25, ~80 kb), and 7A10 (cluster 56, ~90 kb) from top to bottom panel. The first and last lanes are DNA Lambda ladder Markers, the 2nd and 3rd lane(s) on the left hand side of the gels is the control FAC used to transform A. nidulans, and all of other lanes are randomly selected FAC clones recovered. All control and recovered FACs were digested with Not I. Figure S4. Antibiotic activity test of 14 FAC clones. Ten μL out of 150 μL methanol extract from FAC transformants cultured on GMM plate for 7 days at 37°C was loaded on small disc (diameter: 1 cm) for antimicrobial activity test against Aspergillus spp., Candida albicans, Bacillus cereus, Micrococcus luteus and Pseudomonas aeruginosa. Antibiotic activity was observed agai [file 12864_2015_1561_MOESM1_ESM.pdf]

Additional file_1

Fungal artificial chromosome mining of the fungal secondary metabolome

Jin Woo Bok[1,†], Rosa Ye[2,4,†], Kenneth D Clevenger[3,†], David Mead[4], Megan Wagner[4], Amanda Krerowicz[4], Jessica C Albright[5], Anthony W Goering[6], Paul M Thomas[3,6], Neil L Kelleher[3,5,6*], Nancy P Keller[1*], Chengcang C Wu[2,4*]

[1]Department of Medical Microbiology and Immunology and Bacteriology, University of Wisconsin at Madison, Madison, Wisconsin, USA. [2]Intact Genomics, Inc. St Louis, Missouri, USA. [3]Proteomics Center of Excellence, Northwestern University, Evanston, Illinois, USA. [4]Lucigen Corporation, Middleton, Wisconsin, USA. [5]Department of Chemistry, Northwestern University, Evanston, Illinois, USA. [6]Department of Molecular Biosciences, Northwestern University, Evanston, Illinois, USA.

[†]These authors contributed equally to this work.

*Correspondence should be addressed to CCW (cwu@intactgenomics.com), NPK (npkeller@wisc.edu), or NLK (n-kelleher@northwestern.edu)

Supplementary Figures

a

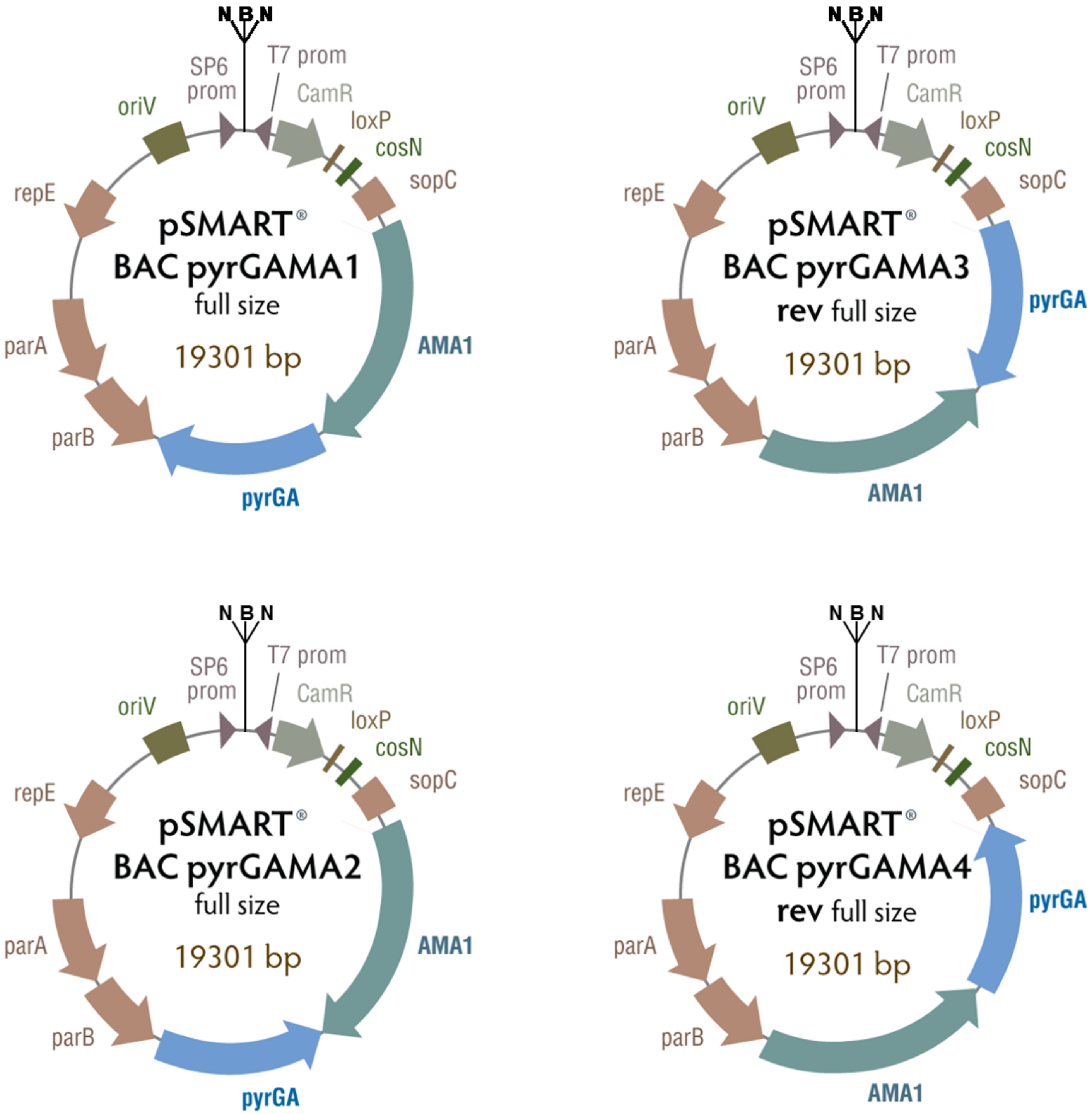

b

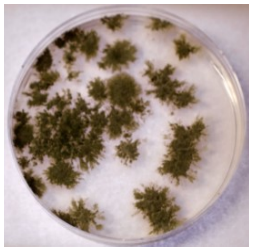

Figure S1. Panel a: FAC vectors: pSMARTBACpyrGAMA1~4, each has two N*ot* I sites (N) flanking the cloning site (B, B*st* XI) within the BAC end sequencing primers SP6 and T7. In addition to the chloramphenicol resistance gene (*camR*), loxP and cos sites, *pyrGA* represents the *pyr*G gene from *Aspergillus parasiticus*, AMA1 is the replication origin of fungal artificial chromosome, genes *par*A and *par*B are for active partitioning and gene *sop*C is to ensure that each daughter cell gets a copy of the shuttle BAC plasmid, gene *rep*E is for BAC plasmid replication and regulation of copy number, and oriV is the BAC replication origin. Panel b: *A. nidulans* FAC transformants using pSMARTBACpyrGAMA3 vector.

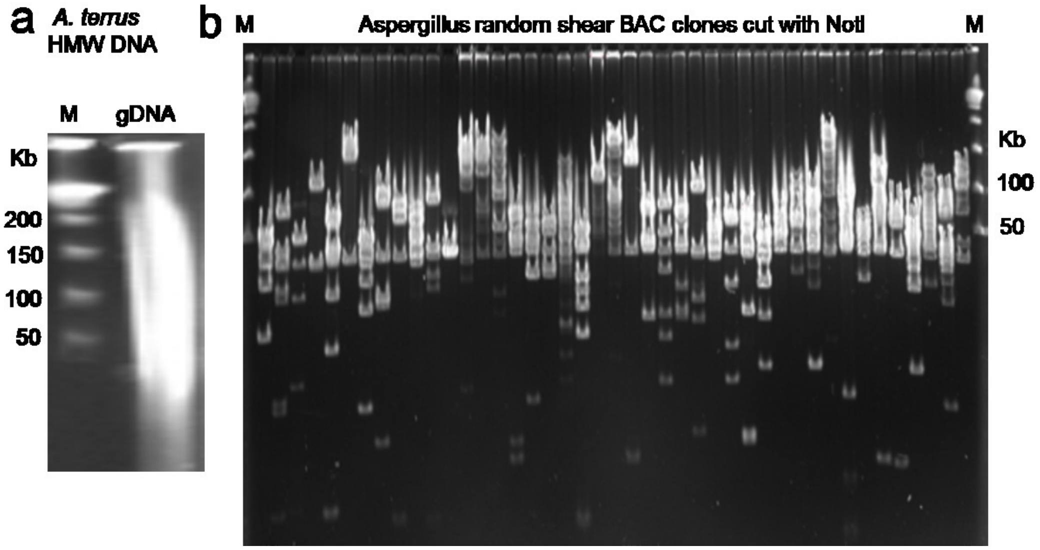

Figure S2. Preparation of HMW genomic DNA from *A. terreus* and random shear FAC cloning results. Panel a: *A. terreus* HMW genomic DNA ranging from 20~200kb. Panel b: CHEF gel electrophoresis and *Not*I digestion of random selected FAC clones, the average insert size was estimated at ~110kb. M, Lambda ladder Marker.

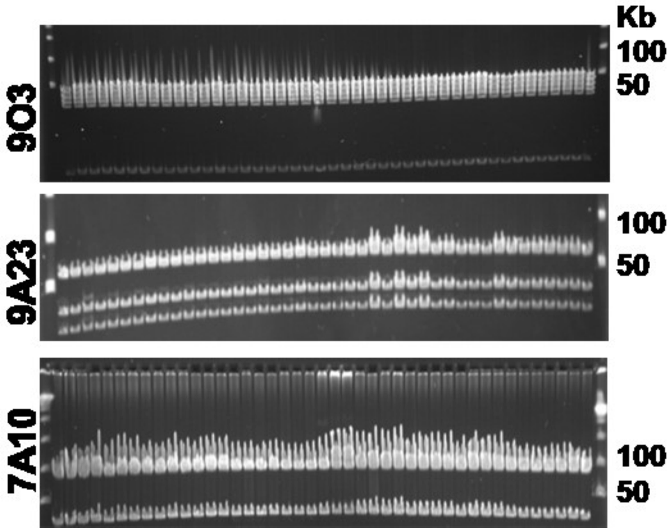

Figure S3. Three additional CHEF gels of *E. coli-Aspergillus* shuttle FACs that were successfully transferred from transformed strains of *A. nidulans* back into *E. coli.* The examples of recovered FAC clones shown here include 9O3 (cluster 30, ~100 kb), 9A23 (cluster 25, ~80 kb), and 7A10 (cluster 56, ~90kb) from top to bottom panel. The first and last lanes are DNA Lambda ladder Markers, the 2nd and 3rd lane(s) on the left hand side of the gels is the control FAC used to transform *A. nidulans*, and all of other lanes are randomly selected FAC clones recovered. All control and recovered FACs were digested with *Not I*.

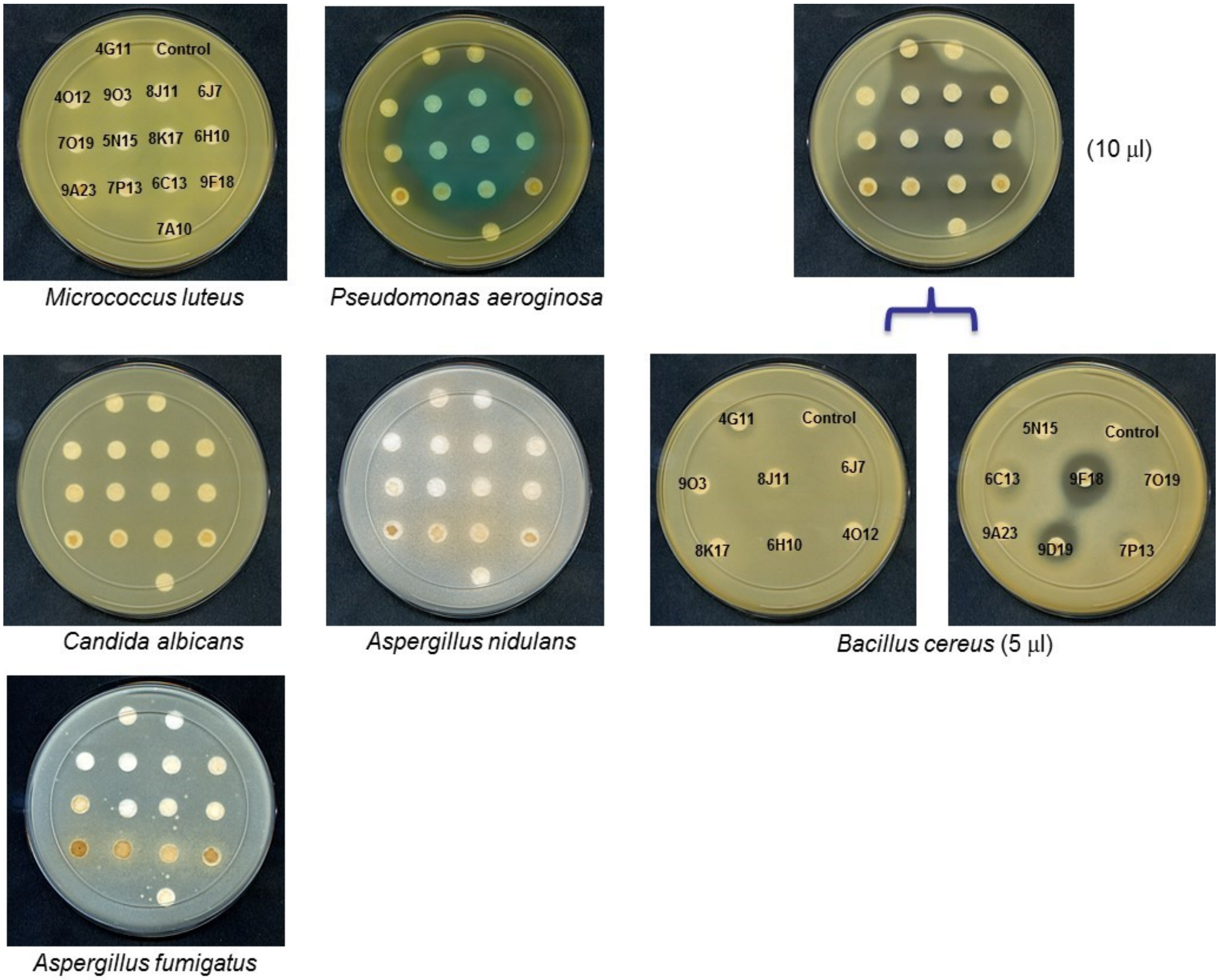

Figure S4. Antibiotic activity test of 14 FAC clones. Ten µl out of 150 µl methanol extract from FAC transformants cultured on GMM plate for 7 days at 37 ^{o}C was loaded on small disc (diameter: 1cm) for antimicrobial activity test against *Aspergillus* spp., *Candida albicans, Bacillus cereus*, *Micrococcus luteus* and *Pseudomonas aeruginosa*. Antibiotic activity was observed against *Bacillus cereus* with two FAC extracts.
