# Supplementary material for: Fungal artificial chromosomes for mining of the fungal secondary metabolome
Source: BMC Genomics. 2015 Apr 29;16(1):343. doi: 10.1186/s12864-015-1561-x (PMC4413528; doi:10.1186/s12864-015-1561-x)
Supplement: Additional file 2: Table S1. — BAC clones covering 56 SM clusters identified by both BAC end sequences. [file 12864_2015_1561_MOESM2_ESM.pdf]

Additional file_2

Fungal artificial chromosome mining of the fungal secondary metabolome

Jin Woo Bok[1,†], Rosa Ye[2,4,†], Kenneth D Clevenger[3,†], David Mead[4], Megan Wagner[4], Amanda Krerowicz[4], Jessica C Albright[5], Anthony W Goering[6], Paul M Thomas[3,6], Neil L Kelleher[3,5,6*], Nancy P Keller[1*], Chengcang C Wu[2,4*]

[1]Department of Medical Microbiology and Immunology and Bacteriology, University of Wisconsin at Madison, Madison, Wisconsin, USA. [2]Intact Genomics, Inc. St Louis, Missouri, USA. [3]Proteomics Center of Excellence, Northwestern University, Evanston, Illinois, USA. [4]Lucigen Corporation, Middleton, Wisconsin, USA. [5]Department of Chemistry, Northwestern University, Evanston, Illinois, USA. [6]Department of Molecular Biosciences, Northwestern University, Evanston, Illinois, USA.

[†]These authors contributed equally to this work.

*Correspondence should be addressed to CCW (cwu@intactgenomics.com), NPK (npkeller@wisc.edu), or NLK (n-kelleher@northwestern.edu)

Supplemental Tables

Table S1. BAC clones covering 56 SM clusters identified by both BAC end sequences.

SM Cluster No.	SM-BAC Clones
1	4H7, 10N10, 4O23, 8P5, 10L6, 3O5
2	9H24, 9K17, 3O16, 9C14
3	10N3, 4P7, 7P21, 6I14, 10B14, 6G18
4	2G12, 9C7, 3K24, 8I15, 6K21, 3P14
5	7C11, 3K10
6	10H6, 9B18, 5G4, 10P8, 8K11, 4A15
7	8P6, 6P5, 7D23, 8L16
8	10G4, 4G18, 5P8, 7G6, 6G20, 10N5
9	3P8, 5L9, 9F19
10	3K24, 1L4, 6H11, 5K16, 9L23
11	4O12,6O11
12	8G17, 6L14, 6F2, 9F16, 3I5
13	5O9, 4L17, 4K8, 4M15, 8L11, 3M21, 5O21, 6C4
14	8M12, 8A13, 3J12, 7D9, 6M9, 10O6, 6O16
15	9P15, 10C14, 9N7, 5J3, 6D6, 3O11
16	9G06, 10O1, 3F16, 5L24, 3N22, 7K5, 5L3
17	3I8
18	6H12, 3F17, 4E23
19	8K17, 5F18, 10J7, 3A20, 3I4, 9D16, 7A13
20	9D11, 8O18, 4G19, 9J14
21	4G11, 7J1, 4O20, 10C20, 8D24, 5M23, 5P9, 3H3, 3F2, 1H24
22	10E11, 8P23, 10F12, 10K23, 6H2, 2M14, 6E8
23	3D6, 9D19, 2E2, 7O23, 3F13
24	10J22, 10E17, 6J17, 6L10, 7K4
25	9A23, 10A2, 6K2, 9A23
26	6K2, 10A2, 10N6, 6E22
27	4O15, 4E15, 7L1, 6I16, 7O24
28	10P13, 5G14, 3G11, 3B22

29	3E2, 6P16, 6J4
30	9O3, 9E16, 6H14, 4E12, 9K13
31	7K15, 9K21, 9M17, 9A1, 4E14, 7E16
32	2M16
33	6A19, 6F11, 10J4, 6K15, 10B8, 5N9
34	1F7, 5M17, 6N10
35	9B9, 3F14, 1L3, 10O14, 8O21
36	9H19, 7I8, 8F13
37	4N23, 8G6, 3L5
38	7O19, 7G7, 6I20, 10J23, 3F9, 3E18
39	5N15, 8P7, 10O23, 9P11, 6D7, 9F12, 9N14, 10C15, 5I22
40	5L7, 4B5, 7P5, 3C6, 8I8
41	6C13, 1H5, 4G24, 9D1, 8A17
42	10E15, 4I9, 5H24
43	6M16, 9A21
44	6J7, 5K20, 5J19, 6H11, 5C17, 9L13
45	3O2, 9I11, 2E10, 1H1
46	5B9, 8N10
47	7J7, 9D10, 4J17, 2C8, 2O20
48	3B4, 6C8, 1H18, 6N20
49	6I22, 9O22, 9N18
50	7P13, 3N1, 5C10, 3N7
51	7M4, 7N2, 7D6, 5N17, 2O2
52	8J19, 3N4, 9J13, 5O19, 7N2, 7D6, 9J13, 5N17
53	6N3, 4A22, 10K22, 7M9
54	3F4, 7A11, 1J21, 10A15, 3M10, 8F11, 6O3, 7M15, 9D9
55	9F18, 10B4, 7G5, 8L22, 8J14, 8A11
56	7A10, 3F5, 4J1, 5N4, 9A3, 8F12, 9K12
