# Supplementary material for: Fungal artificial chromosomes for mining of the fungal secondary metabolome
Source: BMC Genomics. 2015 Apr 29;16(1):343. doi: 10.1186/s12864-015-1561-x (PMC4413528; doi:10.1186/s12864-015-1561-x)
Supplement: Additional file 3: Table S2 — PCR primers used in this study. [file 12864_2015_1561_MOESM3_ESM.pdf]

Additional file_3

Fungal artificial chromosome mining of the fungal secondary metabolome

Jin Woo Bok[1,†], Rosa Ye[2,4,†], Kenneth D Clevenger[3,†], David Mead[4], Megan Wagner[4], Amanda Krerowicz[4], Jessica C Albright[5], Anthony W Goering[6], Paul M Thomas[3,6], Neil L Kelleher[3,5,6*], Nancy P Keller[1*], Chengcang C Wu[2,4*]

[1]Department of Medical Microbiology and Immunology and Bacteriology, University of Wisconsin at Madison, Madison, Wisconsin, USA. [2]Intact Genomics, Inc. St Louis, Missouri, USA. [3]Proteomics Center of Excellence, Northwestern University, Evanston, Illinois, USA. [4]Lucigen Corporation, Middleton, Wisconsin, USA. [5]Department of Chemistry, Northwestern University, Evanston, Illinois, USA. [6]Department of Molecular Biosciences, Northwestern University, Evanston, Illinois, USA.

[†]These authors contributed equally to this work.

*Correspondence should be addressed to CCW (cwu@intactgenomics.com), NPK (npkeller@wisc.edu), or NLK (n-kelleher@northwestern.edu)

Table S2. PCR primers used in this study.

Name	Sequence
**<u>6J7</u>**	
**ATEG_08423**	
8423F	5’TATCTATGCCCTCGGGGTGTGG
8423R	5’AGTCATTGCTGCCCATGAACGGG
**ATEG_08424**	
8424F	5’TCTCCAATCTACTGTCCACCCC
8424R	5’TCTCGATCGGTAAGCGACAAGC
**ATEG_08425**	
8425F	5’TTGGACTGCACTCATGCAAGGC
8425R	5’TTTCTTCAGCAGTTGCGGACC
**ATEG_08426**	
8426F	5’ACAGCAAGGT CCTGCTGTCTGC
8426R	5’TTCAACTGCCACCCAGCCTCC
**ATEG_08427**	
8427F	5’TCTCGGGACTTCAGGTCACTCC
8427R	5’TAACCTTCTGGAACGCGTCTGC
**ATEG_08428**	
08428F	5’ACATATGCCCAACTCGATGCGC
08428R	5’TCCGTCATTCGCAGATGTAGGC
**ATEG_08429**	
8429F	5’TAGTCCACAAACCTACGCTCCG
8429R	5’CACATTACCATTTCCATGGGCG
**ATEG_08430**	
8430F	5’ACGCTATACCAGCAG TCTACCG
8430R	5’AAGGTCCTTTTGGACCTCAGGG
**ATEG_08431**	
8431F	5’ATACGGAGAGACGTGTCCCTCG
8431R	5’ACGTGTTCGTTGAACCCCGAGG
**<u>9D19</u>**	
**ATEG_04319**	
4319F	5’ATTCCAGCAGTACGCTCTGGG
4319R	5’ATGTCTCAATCGCCTCTCGCC
**ATEG_04321**	
4321F	5’AACGCAGAAGAAGGCGACTCG
4321R	5’TTGCCTTTGAGGAGGCCTTCG
**ATEG_04322**	
4322F	5’TCTGGGAGCAGAGTCTCAAGG
4322R	5’TAATCTCCTGCAGAGACCTCTGG
**ATEG_04323**	
4323F	5’TCGGAAACTATAGCGCAGCGG
4323R	5’ACTCGTGGGATCGAGAATCGC

**ATEG_04324**	
4324F	5’TCTGGAAGAAGACGGCGAAGC
4324R	5’ATCCTGTTGCAGGAGGATGCC
**ATEG_04325**	
4325F	5’TCTTTACGGCCTTCACGGTCG
4325R	5’GAGAAAGAGCCAGGATGATGGG
**ATEG_04326**	
4326F	5’ACATCCATGTCTCGCTGACCG
4326R	5’AACTGGATCAGCATCCCACCC
**ATEG_04327**	
4327F	5’AAATCGACAGACTCCCCCAGC
4327R	5’AGGCAAGCCTCATTATCCGGG
**ATEG_04328**	
4328F	5’AGTTGCGATCCATGGTTTCCTCC
4328R	5’TTTTCAGCTCTGCCGAGTCGC
